# Dogs as carriers of virulent and resistant genotypes of *Clostridioides difficile*

**DOI:** 10.1111/zph.12956

**Published:** 2022-05-12

**Authors:** SK Finsterwalder, I Loncaric, A Cabal, MP Szostak, LM Barf, M Marz, F Allerberger, IA Burgener, A Tichy, AT Feßler, S Schwarz, S Monecke, R Ehricht, W Ruppitsch, J Spergser, F Künzel

**Affiliations:** ^1^ Institute of Microbiology University of Veterinary Medicine Vienna Vienna Austria; ^2^ Clinical Unit of Internal Medicine Small Animals University of Veterinary Medicine Vienna Vienna Austria; ^3^ AGES ‐ Austrian Agency for Health and Food Safety Vienna Austria; ^4^ Faculty of Mathematics and Computer Science Friedrich Schiller University Jena Jena Germany; ^5^ Max Planck Institute for Science of Human History Jena Germany; ^6^ FLI Leibniz Institute for Age Research Jena Germany; ^7^ InfectoGnostics Research Campus Jena Jena Germany; ^8^ Department of Biomedical Science University of Veterinary Medicine Vienna Vienna Austria; ^9^ Department of Veterinary Medicine, Centre of Infection Medicine, Institute of Microbiology and Epizootics Freie Universität Berlin Berlin Germany; ^10^ Department of Veterinary Medicine, Veterinary Centre for Resistance Research (TZR) Freie Universität Berlin Berlin Germany; ^11^ Leibniz Institute of Photonic Technology (IPHT) Jena Germany; ^12^ Institut für Medizinische Mikrobiologie und Hygiene Universitätsklinik Dresden Dresden Germany; ^13^ Institute of Physical Chemistry Friedrich Schiller University Jena Jena Germany

**Keywords:** antimicrobial resistance, multilocus sequence typing, One Health, whole‐genome sequencing

## Abstract

While previous research on zoonotic transmission of community‐acquired *Clostridioides difficile* infection (CA‐CDI) focused on food‐producing animals, the present study aimed to investigate whether dogs are carriers of resistant and/or virulent *C. difficile* strains. Rectal swabs were collected from 323 dogs and 38 *C. difficile* isolates (11.8%) were obtained. Isolates were characterized by antimicrobial susceptibility testing, whole‐genome sequencing (WGS) and a DNA hybridization assay. Multilocus sequence typing (MLST), core genome MLST (cgMLST) and screening for virulence and antimicrobial resistance genes were performed based on WGS. Minimum inhibitory concentrations for erythromycin, clindamycin, tetracycline, vancomycin and metronidazole were determined by E‐test. Out of 38 *C. difficile* isolates, 28 (73.7%) carried genes for toxins. The majority of isolates belonged to MLST sequence types (STs) of clade I and one to clade V. Several isolates belonged to STs previously associated with human CA‐CDI. However, cgMLST showed low genetic relatedness between the isolates of this study and *C. difficile* strains isolated from humans in Austria for which genome sequences were publicly available. Four isolates (10.5%) displayed resistance to three of the tested antimicrobial agents. Isolates exhibited resistance to erythromycin, clindamycin, tetracycline and metronidazole. These phenotypic resistances were supported by the presence of the resistance genes *erm*(B), *cfr*(C) and *tet*(M)*.* All isolates were susceptible to vancomycin. Our results indicate that dogs may carry virulent and antimicrobial‐resistant *C. difficile* strains.

**Impacts**

- This study underlines once again the assumption that dogs may be carriers of zoonotic *Clostridioides difficile* strains. Since asymptomatic carriage is likely in dogs, they might be an unknown reservoir from which *C. difficile* may disseminate. Thus, we consider in our study a One Health approach connecting human and veterinary medicine, which are both equally important to combat life‐threatening *C. difficile* infections. Here we provide a basis for comparison of *C. difficile* isolates based on whole‐genome sequencing.

## 1 INTRODUCTION

*Clostridioides difficile* infections (CDI) are major causes of antibiotic‐associated diarrhoea in healthcare settings worldwide. In Europe, CDI account for 44.6% of gastrointestinal healthcare‐associated infections (Suetens et al., 2018). In addition to healthcare‐associated CDI, community‐acquired *C. difficile* infections (CA‐CDI) have also been studied recently. Previous studies pointed to pets as potential sources of CA‐CDI (Rabold et al., 2018; Stone et al., 2016). In healthy dogs, *C. difficile* presence ranges between 3.4% and 30% (Hussain et al., 2015; Rabold et al., 2018; Schneeberg et al., 2012; Stone et al., 2016; Usui et al., 2016; Weese et al., 2019). Although toxigenic and non‐toxigenic isolates were detected in dogs, including the epidemic hypervirulent PCR ribotype (RT) 027 (Rabold et al., 2018), genomic information on *C. difficile* strains isolated from animals other than livestock and horses is scarce (Knight et al., 2019; Knight & Riley, 2019; Rodriguez et al., 2016). Furthermore, methods for genetic comparison with high discriminatory power, reproducibility and transferability are readily available nowadays, e.g. multilocus sequence typing (MLST), core genome MLST (cgMLST), single‐nucleotide polymorphism or multilocus variable number of tandem repeat analysis (Bletz et al., 2018; Griffiths et al., 2010; Knetsch et al., 2013; Marsh et al., 2006), but data obtained from the application of the aforementioned methods are still limited in studies of canine *C. difficile* isolates.

Moreover, RT078 is emerging amongst *C. difficile* strains and is associated – amongst other RTs – with MLST sequence type (ST)11 (Knight et al., 2019). This RT is particularly often isolated from cases of CA‐CDI (Knight & Riley, 2019), is widespread in food‐producing animals (Knight & Riley, 2019) and international transmission of ST11 has been described (Knight et al., 2019). RT078 has also been detected in dogs (Orden et al., 2017; Rabold et al., 2018).

Currently, only a small number of studies on antimicrobial resistance (AMR) of *C. difficile* recovered from dogs has been performed, which reported reduced susceptibility of *C. difficile* isolates against metronidazole, erythromycin, clindamycin, β‐lactam antibiotics and fluoroquinolones (Orden et al., 2017; Spigaglia et al., 2015; Usui et al., 2016). At the genetic level, the resistance genes *erm*(B) and *tet*(M) were identified in human isolates (Spigaglia, 2016), whereas the underlying resistance mechanisms in canine isolates were only rarely assessed. Recent reports support the assumption that asymptomatic carriage of *C. difficile* in dogs is more likely to occur than gastrointestinal disease (Busch et al., 2015; Stone et al., 2016, 2019). Therefore, dogs might serve as a hidden reservoir of virulent and resistant *C. difficile* strains.

The aims of the present study were (a) to assess the prevalence of dogs colonized with *C. difficile* in the city of Vienna, Austria and in the surrounding area; (b) to characterize and compare the obtained *C. difficile* isolates using antimicrobial susceptibility testing, whole‐genome sequencing (WGS) and a DNA hybridisation assay and finally (c) to examine associations between *C. difficile* and enteric disease in dogs and potential risk factors for shedding.

## 2 MATERIALS AND METHODS

### 2.1 Sampling and ethics

Two rectal swabs were taken simultaneously from each of 323 dogs. Diseased dogs were patients of the small animal clinic at the Vetmeduni Vienna, whereas samples from healthy dogs were provided from colleagues, students, dog trainers and dog sitters. A questionnaire regarding signalment and anamnesis was completed by all dog owners. Approval of this study was obtained from the ethics committee of Vetmeduni Vienna (ETK 02/02/17). All dog owners provided a written informed consent.

### 2.2 Isolation and identification of *Clostridioides difficile*

Two methods for specimen cultivation were performed. The first swab was directly plated after application of the alcohol shock method as described in a previous protocol with minor modifications (Blanco et al., 2013). In contrast to Blanco et al. (2013), the swab tip was left in the sealed microtubes and incubated at room temperature for 20 min. Samples were then plated onto BD™ Columbia Agar with 5% sheep blood (BA) or BD™ Columbia CNA Agar with 5% sheep blood, Improved II (Becton Dickinson). Plates were incubated for 48–72 hr at 37°C under anaerobic conditions.

The second swab underwent selective enrichment as documented (Blanco et al., 2013). The alcohol shock method (Arroyo et al., 2005) was applied once after 2–3 days (two samples after 4 days) and once after 7–8 days (two samples after 9 days) of anaerobic incubation by mixing 1 ml of broth culture with an equal volume of absolute ethanol. Alcohol‐shocked samples were plated onto BA and incubated as described above.

Colonies with typical *C. difficile* morphology were identified to species level by matrix‐assisted laser desorption/ionization‐time‐of‐flight (MALDI‐TOF) mass spectrometry (Bruker, Daltonik). One colony representing each a distinct colony morphotype was sub‐cultivated on BA and incubated as described above to obtain a pure culture. Isolates were cryo‐conserved at −80°C until further analysis.

### 2.3 Antimicrobial susceptibility testing

Bacterial suspensions and MIC gradient stripes containing erythromycin, vancomycin, metronidazole, tetracycline and clindamycin (bioMérieux, Marcy l'Etoile, France; Oxoid; Himedia) were plated onto BD™ Brucella Blood Agar with Hemin and Vitamin K1 (Becton Dickinson) following the manufacturer's instructions. According to the recommendations of the Clinical and Laboratory Institute (CLSI), the following breakpoints for resistance were used: erythromycin and clindamycin 8 mg/L, tetracycline 16 mg/L, metronidazole 32 mg/L (CLSI, 2021; Spigaglia et al., 2011). CLSI‐approved clinical breakpoints for vancomycin were not available. The reference strain *C. difficile* ATCC®700057 was used as quality control strain.

### 2.4 Whole‐genome sequencing and comparative genomic analysis

Bacterial DNA was extracted using MagAttract HMW DNA Kit (Qiagen). Ready‐to‐sequence libraries were prepared using Nextera XT DNA Library Preparation Kit (Illumina). Paired‐end WGS was conducted in an Illumina MiSeq platform (Illumina Inc.). Raw reads were de novo assembled using SPAdes assembler 3.11.1 (Bankevich et al., 2012). The quality of the assembled genomes was evaluated using QUAST v5.0.2 (Gurevich et al., 2013). For annotation of coding sequences, the command line software tool Prokka v1.13.4 (Seemann, 2014) was applied. Species were determined with JSpecies workspace using ANIb (average nucleotide identity via BLAST) analysis tool (Richter et al., 2015). In silico MLST (Griffiths et al., 2010) and cgMLST (Bletz et al., 2018) were carried out with SeqSphere + software 5.0.4‐rc03_(2018–05) (Ridom). A minimum spanning tree based on cgMLST data was constructed to investigate genetic relatedness between the isolates obtained in this study and 10 publicly available *C. difficile* genomes from different origins in Austria (Table S1). Of the latter, seven were downloaded from Enterobase (https://enterobase.warwick.ac.uk/species/index/clostridium) (Frentrup et al., 2020) and three were retrieved from a previously published work (Eyre et al., 2018). Isolates were considered to be clonally related when differing by ≤6 alleles in cgMLST (Bletz et al., 2018). In addition, contigs were screened for virulence genes (*cdtA*, *cdtB*, *tcdA*, *tcdB*, *tcdC*). Sequenced isolates were used for in silico prediction of hybridization patterns on a previously developed microarray system (Gawlik et al., 2015). Isolates were then assigned to pre‐described hybridization profiles (HP) (Gawlik et al., 2015). The Comprehensive Antibiotic Resistance Database (CARD; https://card.mcmaster.ca/home) (Alcock et al., 2020) as well as ResFinder 4.1 (https://cge.cbs.dtu.dk/services/ResFinder/) (Bortolaia et al., 2020) were used for identification of chromosomal mutations and/or acquired resistance genes in isolates exhibiting increased MIC values. Furthermore, Geneious Prime February 2, 2021 software served for sequence alignment and BLAST analyses of WGS data of the metronidazole resistant isolate to the recently described plasmid pCD‐METRO (Boekhoud et al., 2020).

### 2.5 Statistical analysis

Correlations between shedding of *C. difficile* and different risk factors for shedding were assessed by using the χ^2^‐test (statistical significance with *p*‐values < .05). Investigated risk factors were age, gender, illness, medical profession of the owner, close owner contact, type of feeding, history of acute and/or chronic diarrhoea, previous antibiotic treatment (within the past 4 weeks), visits to the vet and immunosuppression (within the past 6 months). In addition, Odds Ratios with 95% confidence interval were calculated.

## 3 RESULTS

### 3.1 Prevalence of *Clostridioides difficile* and risk factors for shedding

In total, 38 isolates were recovered from 38 of the 323 examined dogs (11.8%). One isolate (2.6%) was recovered solely by direct plating and the remaining 37 isolates (97.4%) were detected after direct plating and/or enrichment (Table S2).

Analysis of the dogs' specific anamnestic data (Table S3) revealed no association between shedding of *C. difficile* and the queried risk factors. However, dogs which were or had been recently patients at the intensive care unit (ICU) of Vetmeduni Vienna were more likely carrier of *C. difficile* (33.3%). Previous antimicrobial treatment in the past 4 weeks prior to sampling led to higher colonization rates (26.4%) in comparison to dogs without previous antimicrobial therapy (8.9%). This observation is underlined by comparing the data of *C. difficile* positive dogs pre‐treated with one (20.0%) or multiple antimicrobial agents (38.5%). Moreover, dogs regularly visiting one specific dog sitter had a higher chance to become colonized with *C. difficile* (53.8%; data not shown). However, none of these observations was statistically significant.

### 3.2 Antimicrobial susceptibility testing and detection of antimicrobial resistance determinants

Resistance to erythromycin (MIC > 256 mg/L), clindamycin (MIC > 256 mg/L), tetracycline (MIC = 24–64 mg/L) and metronidazole (MIC = 32 mg/L) was found in seven (18.4%), eleven (28.9%), six (15.8%) and one (2.6%) isolate, respectively (Table 1). All isolates exhibited MIC values of ≤1 mg/L to vancomycin and can, therefore, be considered as susceptible to vancomycin. Four isolates (10.5%) displayed resistance to three of the tested antimicrobial agents. Isolates with resistance to erythromycin carried the macrolide resistance gene *erm*(B) (*n* = 4), a mutation C → T at position 656 of the 23S rDNA (C656T) (*n* = 1, 14.3%) or both the *erm*(B) and the C656T mutation (*n* = 2, 28.6%; Table 1). This mutation of 23S rDNA was found in 25 isolates (65.8%) in total (data not shown), of which 22 isolates (88.0%) were susceptible to erythromycin. The genes *erm*(B) (*n* = 6, 54.5%) and *cfr*(C) (*n* = 1, 9.1%) were detected in isolates with resistance to clindamycin. In four isolates (36.4%) with clindamycin MIC values of >256 mg/L, no known resistance mechanism was detected (Table 1). In isolates with tetracycline resistance, the gene *tet*(M) (*n* = 3, 50.0%) or no known resistance mechanism (*n* = 3, 50.0%) was detected (Table 1). In addition, the isolate showing resistance to metronidazole recovered in our study harboured the 7056‐bp plasmid pCD‐METRO (Table 1).

**TABLE 1:** Isolates of *Clostridioides difficile* in this study (*n* = 38)

ID	MLST		HP	Antimicrobial resistance profile						Toxin genes
	Clade	ST		Phenotype (MIC [mg/L])					Genotype	Genes PaLoc
				ERY	CLI	TET	VAN	MTZ		
				≥8	≥8	≥16	n.a.	≥32		
35^a^	I	2^†^	HP‐13	0.75	3	0.064	0.5	0.064		*tcdA*, *tcdB*, *tcdC*
38			HP‐13	1.5	3	0.38	0.5	0.125		*tcdA*, *tcdB*, *tcdC*
39^a^			HP‐13	1.5	3	0.064	0.5	0.023		*tcdA*, *tcdB*, *tcdC*
47			HP‐13	0.75	3	0.047	0.5	0.047		*tcdA*, *tcdB*, *tcdC*
50			HP‐13	1.5	0.75	0.094	0.5	0.047		*tcdA*, *tcdB*, *tcdC*
55			HP‐13	0.75	3	0.19	0.5	0.032		*tcdA*, *tcdB*, *tcdC*
97^§^			New	1	2	0.38	0.5	0.032		*tcdA*, *tcdB*, *tcdC*
99			HP‐13	1.5	3	0.38	0.25	0.064		*tcdA*, *tcdB*, *tcdC*
171^b^			HP‐13	1	2	0.38	0.5	0.064		*tcdA*, *tcdB*, *tcdC*
251			HP‐13	1	0.75	0.19	0.5	0.064		*tcdA*, *tcdB*, *tcdC*
272^c^			HP‐13	0.75	2	0.023	0.5	0.032		*tcdA*, *tcdB*, *tcdC*
273^c^			HP‐13	1.5	1	0.064	0.5	0.032		*tcdA*, *tcdB*, *tcdC*
279			New	1	3	0.25	0.5	0.064		*tcdA*, *tcdB*, *tcdC*
333			HP‐13	0.75	1	0.047	0.5	0.047		*tcdA*, *tcdB*, *tcdC*
108		110	HP‐13	1	1.5	0.06	0.5	0.064		*tcdA*, *tcdB*, *tcdC*
164^b^			HP‐13	1	4	0.38	0.5	0.094		*tcdA*, *tcdB*, *tcdC*
157		42^†^	HP‐13	0.38	**>256**	**64**	0.5	0.125		*tcdA*, *tcdB*, *tcdC*
225			HP‐13	1	**>256**	**24**	0.25	0.064		*tcdA*, *tcdB*, *tcdC*
345			HP‐13	0.75	6	0.125	0.25	0.125		*tcdA*, *tcdB*, *tcdC*
62		3^†^	HP‐01	**>256**	**>256**	4	0.5	0.032	*erm*(B)	*tcdA*, *tcdB*, *tcdC*
37			HP‐11	1	0.75	0.125	1	0.125		
161^§^			HP‐11	0.5	2	0.25	0.5	0.032		
308			HP‐11	0.75	3	0.19	0.5	0.047		
142^§^		107^‡^	HP‐11	0.38	**>256**	0.125	1	0.032		
112^§^		15^†^	HP‐27	**>256**	**>256**	0.125	0.5	0.047	*erm*(B)	
241			HP‐27	**>256**	**>256**	0.016	1	**32**	*erm*(B), pCD‐METRO	
321			HP‐27	**>256**	**>256**	0.016	1	0.047	*cfr*(C)	
337			HP‐27	1	2	0.032	0.5	0.094		
54		26	HP‐26	1	3	1	1	0.094		
316			HP‐26	1.5	3	4	0.25	0.047		
163^b^		54^†^ ^,^ ^‡^	New	**>256**	**>256**	**32**	0.5	0.047	*erm*(B), *tet*(M)	*tcdA*, *tcdB*, *tcdC*
169^b^			n.t.	**>256**	**>256**	**64**	0.5	0.032	*erm*(B), *tet*(M)	*tcdA*, *tcdB*, *tcdC*
170^b^			n.t.	**>256**	**>256**	**48**	0.5	0.023	*erm*(B), *tet*(M)	*tcdA*, *tcdB*, *tcdC*
140^d^		236^‡^	New	1	6	0.25	0.5	0.032		*tcdA*, *tcdB*, *tcdC*
141^d^			New	0.75	**>256**	**32**	0.5	0.064		*tcdA*, *tcdB*, *tcdC*
173^b^		239^‡^	New	0.5	4	0.25	0.5	0.032		*tcdA*, *tcdB*, *tcdC*
174^b^			New	0.5	3	0.032	0.5	0.032		*tcdA*, *tcdB*, *tcdC*
223	V	11^†^ ^,^ ^‡^	HP‐37	0.38	2	6	0.5	0.016		*tcdA*, *tcdB*, *tcdC* ^¶^
In total		7	11	6	0	1		28		

Sample numbers (ID), MLST clade and sequence types (ST), microarray hybridization profiles (HP), antimicrobial resistance profile with phenotype and breakpoints if available based on CLSI (minimum inhibitory concentration (MIC) of erythromycin (ERY), clindamycin (CLI), tetracycline (TET), vancomycin (VAN) and metronidazole (MTZ)), genotype (resistance genes, mutations and specific plasmid) and genes of pathogenicity locus (PaLoc: *tcdA*, *tcdB*, *tcdC*) of detected isolates. Isolates from dogs living in the same household (a, c and d) and visiting the same dog sitter (b) are marked with letters. Resistant MIC values are marked in bold and summed at end of the table.

Abbreviations: n.a., not available; n.t., non‐typeable.

^†^ ST associated with commonly isolated RTs in CDI in Europe and Austria.

^‡^ ST previously not described in dogs to our knowledge.

^§^ isolates from dogs with recent stay at the ICU of Vetmeduni Vienna.

^¶^ *tcdC* with nonsense mutation.

### 3.3 Genomic characterization of canine *Clostridioides difficile*

**FIGURE 1:**
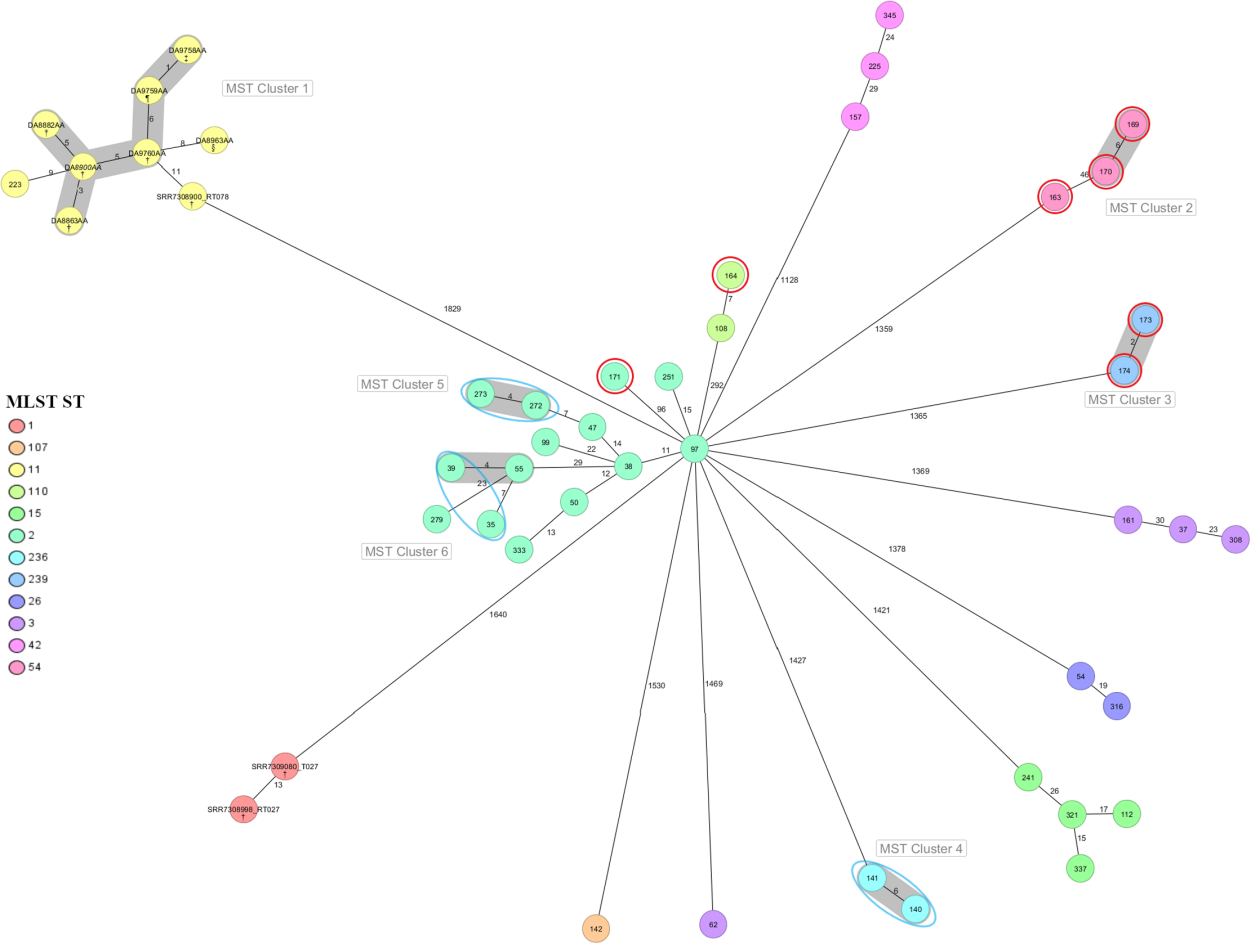
cgMLST minimum spanning tree of the detected 38 isolates in this study compared to isolates collected in Austria, generated with Ridom SeqSphere+, based on 2270 columns, pairwise ignoring missing values; sample ID in circles, different MLST STs colour‐marked and signified in legends; clonal complex designated as cluster 1‐6 (cluster distance threshold: 6) and highlighted in light grey; isolates from dogs living in the same household encircled red, isolates from dogs visiting the same dog sitter marked with blue circles. †Human isolates, ‡porcine isolates, §bovine isolates, and isolate with unknown origin

The minimum spanning tree shows grouping of the *C. difficile* isolates by their STs and six clonally related clusters were identified (Figure 1). Five of those clusters included 10 of our isolates and the remaining 28 isolates were found as singletons. Eleven distinct STs were identified amongst the 38 isolates with ST2 being the most prevalent (*n* = 14, 36.8%) (Table 1). All but one of the canine *C. difficile* isolates were assigned to clade I. The genetically distant outlier was allocated to ST11, belonging to clade V, and did not group with any other of our isolates. Based on cgMLST, it differed by only nine alleles with isolates in cluster 1, which included human and porcine isolates downloaded from Enterobase (Figure 1). Clusters 2 to 5 were composed by isolates collected from dogs who had contact with other dogs (Figure 1). Only one cluster (cluster 6) included isolates from dogs with no contact to other dogs.

Hybridization profiles of the *C. difficile* isolates revealed six distinct pre‐described HPs (Gawlik et al., 2015) with HP‐13 (44.7%) being most frequently detected (Table 1). Allocation to previously known HPs was not possible for seven isolates and two isolates were non‐typeable. HPs coincided mainly with STs, but some STs clustered together in the same HPs (Table 1).

The genes for pathogenicity locus (*tcdB*, *tcdA*, *tcdC*) were detected in 28 isolates (73.7%) (Table 1), whereas only the ST11 isolate harboured genes for binary toxin (CDT) (*cdtA, cdtB*; *n* = 1; 2.6%). ST11 also showed a *tcdC* allele that produces a severe truncated TcdC protein due to a nonsense mutation at position 184.

Some isolates harbouring antimicrobial resistance mechanisms were associated with distinct STs (Table 1). Isolates of ST54 (*n* = 3, 100%), ST15 (*n* = 2, 50.0%) and the toxigenic ST3 isolate (*n* = 1, 100%) carried the gene *erm*(B). All isolates of ST54 (*n* = 3, 100%) also harboured *tet*(M) and were resistant to tetracycline.

All genome sequences determined in this study are available under BioProject Number PRJNA789842 in GenBank and ID 1838 in the PubMLST database (https://pubmlst.org/) (Griffiths et al., 2010).

### 3.4 Genome annotation and comparison

Genomic features of assembled isolates are summarized in Table S4. The genome sizes of the *C. difficile* isolates ranged as expected from 4.00 Mb (ID 157) to 4.63 Mb (ID 108). The numbers of assembled contigs of isolated strains ranged from 183 (ID 345) to 3141 (ID 108) contigs. The GC content varied between 28.52% (ID 112) and 31.27% (ID 108). The numbers of predicted coding sequences (CDS) ranged from 3458 (ID 157) to 4051 (ID 142).

## 4 DISCUSSION

The overall prevalence of *C. difficile* (11.8%) in dogs and the percentage of toxigenic isolates (73.7%) found in our study are consistent with previous findings (Hussain et al., 2015; Rabold et al., 2018; Stone et al., 2016; Usui et al., 2016; Weese et al., 2010, 2019). Prior antibiotic treatment was not a significant risk factor for shedding *C. difficile*, which was in contrast to other authors' findings (Clooten et al., 2008; Rabold et al., 2018). Colonization of pets during hospitalization has already been demonstrated (Clooten et al., 2008), which was supported by the data of the present study showing higher colonization rates of dogs hospitalized at the ICU. Furthermore, our results are in agreement with the previously discussed assumption that dogs are predominantly asymptomatic carriers of *C. difficile* (Busch et al., 2015; Stone et al., 2016, 2019).

*Clostridioides difficile* has shown to accumulate and spread antimicrobial resistance genes via intra‐ and interspecies horizontal gene transfer (Corver et al., 2012; Knight et al., 2019; Knight & Riley, 2019; Spigaglia, 2016), and a highly mosaic and dynamic structure of the *C. difficile* genome has been demonstrated (Sebaihia et al., 2006). Eleven isolates (28.9%) analysed in this study exhibited resistance to at least one of the tested antimicrobial agents. Consequently, the canine gut microbiota might serve as a reservoir for AMR genes. This is of particular interest if occurrence of interspecies transmission of *C. difficile* is assumed.

One limitation inherent to this study was that we could not clarify the genetic basis for some of the observed resistances, e.g. for erythromycin (*n* = 1, 14.3%), clindamycin (*n* = 4, 36.4%) and tetracycline (*n* = 3, 50.0%) (Table 1). The C656T mutation, which represents a target mutation, was thought to mediate high‐level resistance to erythromycin and low‐level resistance to clindamycin when first discovered (Schmidt et al., 2007). However, this mutation has also been found in susceptible strains (Spigaglia et al., 2011), thereby questioning its role in AMR. The multidrug resistance gene *cfr*(C), which has only been recently described in *C. difficile* (Candela et al., 2017), modifies the ribosomal binding site of phenicols, lincosamides, oxazolidinones, pleuromutilins and streptogramin A (PhLOPS_A_). Therefore, the presence of *cfr*(C) might be responsible for clindamycin resistance.

Resistance mechanisms for metronidazole in *C. difficile* are still not fully understood (Spigaglia, 2016) and, therefore, a topic of current research. Very recently, a new plasmid (pCD‐METRO) has been identified that confers resistance to metronidazole (Boekhoud et al., 2020). Amongst the eight open reading frames (ORFs) of pCD‐METRO, ORF6 encodes for a protein with homology to NimB of *Bacteroides fragilis*, a 5‐nitroimidazole reductase (Boekhoud et al., 2020). So far the plasmid has been detected in *C. difficile* isolates of human and animal origin from Austria (our study), Spain, Germany, Poland and the Czech Republic (Boekhoud et al., 2020). Therefore, it seems plausible that pCD‐METRO is internationally disseminated, at least in Europe.

Information on genetic relatedness of canine *C. difficile* is scarce. Our data support the assumption of intraspecies transmission (Schneeberg et al., 2012) since dogs that had contact to each other shared the same ST. Moreover, some formed clonal complexes. On the other hand, dogs visiting the same dog sitter showed shedding of various STs, which could be explained by co‐colonization of multiple STs in one host (Stone et al., 2016) and by our approach to analyse only one colony of the primary culture. For further investigations, we suggest to subculture and investigate more than one colony representing a distinct colony morphotype to assess potentially different strains harboured by a single host. Moreover, cluster 6 included isolates from dogs with no direct contact to each other. However, there could be a possible transmission path, because the owners of these dogs met each other at work.

A previous report found 12 distinct STs in dogs, all belonging to the non‐hypervirulent clade I (Stone et al., 2016). Our results underscore these findings, since the majority of our isolates (97.4%) belonged to clade I. Three STs (2, 3, 42) have been frequently isolated from cases of CDI in humans (Dingle et al., 2011). ST2 has been associated with RT014 and RT020 amongst others and ST42 with RT106, i.e. with ribotypes that are commonly detected in human patients with CDI in Austria (Nationale Referenzzentrale für Clostridioides difficile, 2019).

To our knowledge, toxigenic ST54, ST236, ST239 and non‐toxigenic ST107 have not been isolated from dogs up to now. The finding of ST54 is noteworthy, since it has been associated with RT012 (Griffiths et al., 2010), a ribotype regularly isolated from CDI of humans in Europe (Freeman et al., 2020). Since the data on STs distribution in canine isolates is scarce, ST11 has not been described in dogs so far. However, in two studies, RT078 was found in dogs (Orden et al., 2017; Rabold et al., 2018), which is associated with ST11 amongst other STs (Griffiths et al., 2010; Knight et al., 2019). ST11 differed from other detected STs in this study in all seven MLST loci and is therefore solely categorized in clade V. Detection of ST11 in dogs is of public health concern, since a pan‐European surveillance recently showed that RT078 was amongst the five most frequently occurring ribotypes in CDI (Freeman et al., 2020) and ST11 is of public health concern worldwide (Knight et al., 2019). This emerging ST showed increased pathogenicity in comparison to other STs, and it is known to frequently affect younger patients being considered as an important cause of CA‐CDI (Knight & Riley, 2019). It was hypothesized that a truncated TcdC protein, which is associated with ST11 (Knight et al., 2019), may contribute to the increased pathogenicity due to a higher level of toxin production of these strains (Spigaglia & Mastrantonio, 2002). Although, the detected ST11 isolate did not form a clonal complex with the isolates in cluster 1, the number of allelic differences (nine alleles to the nearest human strain) was low considering the absence of epidemiological relationship between them.

Finally, DNA‐microarray typing provides a rapid genotyping method to characterize clinical *C. difficile* isolates from patient samples (Gawlik et al., 2015). Clustering of assigned HPs overlapped largely with associated STs in this study as expected (Gawlik et al., 2015). Furthermore, the single toxigenic ST3 isolate showing great distance in cgMLST to the other ST3 isolates was assigned to a distinct HP. This supports the presumption that array hybridization produces comparable phylogenetic information like MLST and other typing methods (Gawlik et al., 2015). On the other hand, STs which matched in six (ST2 and ST110), five (ST2 and ST42) or four MLST loci (ST3 and ST107) shared the same HP and ST3 and ST42 were allocated to different HPs, despite differing only in one MLST locus. Nevertheless, microarray assay can be a useful tool to compare in vitro typing data to sequence data, the latter readily accessible via databases.

In conclusion, our data support the hypothesis that dogs can be carriers of virulent and antimicrobial‐resistant *C. difficile* strains. Considering that asymptomatic carriage is frequent in dogs, the transmission potential of *C. difficile* via companion animals should not be underestimated. Especially, the presence of human‐associated STs in dogs is an issue of concern, since this may point towards an exchange of *C. difficile* in either direction. Control of CDI can only be achieved through a One Health approach, including human and veterinary medicine as well as expanding the focus to non‐food‐producing animals.

## CONFLICT OF INTEREST

The authors do not have any conflicts of interest to declare.

## Supporting information


Table S1

Table S2

Table S3

Table S4

## Data Availability

All genome sequences determined in this study are available under BioProject Number PRJNA789842 in Gen Bank and ID 1838 in the PubMLST database.
