# Supplementary material for: Dogs as carriers of virulent and resistant genotypes of Clostridioides difficile
Source: Zoonoses Public Health. 2022 May 12;69(6):673–81. doi: 10.1111/zph.12956 (PMC9544694; doi:10.1111/zph.12956)
Supplement: Supplementary file 1 — Table S1 [file ZPH-69-673-s004.pdf]

Table S1 Accession numbers of publicly available *C. difficile* genomes used in this study

ID	origin	accession number	BioProject	publication source
DA8882AA	human	SAMEA1710506	PRJEB3012	Enterobase (https://enterobase.warwick.ac.uk/species/index/clostridium)
DA9760AA		SAMEA1710492		
DA8863AA		SAMEA1710543		
DA8900AA		SAMEA1710461		
DA9758AA	porcine	SAMEA1710314		
DA8963AA	bovine	SAMEA1710318		
DA9759AA	n.a.	SAMEA1710536		
SRR7309080	human	SAMN09406834	PRJNA398458	Eyre et al., 2018
SRR7308998		SAMN09406342		
SRR7308900		SAMN09406759		

Genome number (ID); n.a. = not available

References

Eyre, D. W., Davies, K. A., Davis, G., Fawley, W. N., Dingle, K. E., De Maio, N., Karas, A., Crook, D. W., Peto, T. E. A., Walker, A. S., & Wilcox, M. H. (2018). Two Distinct Patterns of *Clostridium difficile* Diversity Across Europe Indicating Contrasting Routes of Spread. *Clinical Infectious Diseases*, **67**, 1035–1044. https://doi.org/10.1093/cid/ciy252
