# Supplementary material for: Dogs as carriers of virulent and resistant genotypes of Clostridioides difficile
Source: Zoonoses Public Health. 2022 May 12;69(6):673–81. doi: 10.1111/zph.12956 (PMC9544694; doi:10.1111/zph.12956)
Supplement: Supplementary file 2 — Table S2 [file ZPH-69-673-s002.pdf]

Table S2 Outcome of used isolation methods for *C. difficile*

ID	direct	enr 1	enr 2
35†	neg	pos	pos
37	pos	pos	pos
38	pos	pos	pos
39†	neg	neg	pos
47	neg	neg	pos
50	neg	neg	pos
54	neg	neg	pos
55	neg	pos	pos
62	neg	pos	pos
97	pos	pos	pos
99	neg	neg	pos
108	neg	pos	pos
112	neg	pos	pos
140	neg	neg	pos
141	neg	neg	pos
142	neg	neg	pos
157	neg	neg	pos
161	neg	neg	pos
163	neg	neg	pos
164	neg	neg	pos
169	neg	neg	pos
170	neg	neg	pos
171	neg	neg	pos
173	neg	neg	pos
174	neg	neg	pos
223	neg	neg	pos
225	neg	neg	pos
241	neg	pos	pos
251	pos	neg	neg
272	neg	pos	pos
273	neg	neg	pos
279	neg	pos	pos
308	neg	pos	pos
316	pos	pos	pos
321	neg	neg	pos
333	neg	pos	pos
337	neg	pos	pos
345	neg	pos	pos
**In total**	5	16	37

Direct plating after alcohol shock method (direct) and plating after alcohol shock method performed after 2-3 days (enr 1) and 7-8 days (enr 2) of enrichment. Confirmation for positive (pos) and negative (neg) samples with MALDI TOF. †alcohol shock and plating performed after 4 instead after 2-3 days in enr 1.
