# Supplementary material for: Dogs as carriers of virulent and resistant genotypes of Clostridioides difficile
Source: Zoonoses Public Health. 2022 May 12;69(6):673–81. doi: 10.1111/zph.12956 (PMC9544694; doi:10.1111/zph.12956)
Supplement: Supplementary file 3 — Table S3 [file ZPH-69-673-s003.pdf]

Table S3 Questionnaire data of sampled dogs

criteria	group and subgroup		Total numbers		Numbers of *C. difficile* positive	
age	<1		22	(6.8%)	1	(4.5%)
	1-8		165	(51.1%)	16	(9.7%)
	>8		136	(42.1%)	21	(15.4%)
gender	female		182	(56.3%)	22	(12.1%)
		female intact	54	(16.7%)	5	(9.3%)
		female neutered	128	(39.6%)	17	(13.3%)
	male		141	(43.7%)	16	(11.3%)
		male intact	45	(13.9%)	2	(4.4%)
		male neutered	92	(28.5%)	14	(2.5%)
		male chemical castrated	4	(1.2%)	0	(0.0%)
Source of sample	healthy dogs		187	(57.9%)	19	(10.2%)
	patient of Vetmeduni		136	(42.1%)	19	(14.0%)
Profession of owner†	veterinarian		40	(12.4%)	1	(2.5%)
	veterinary technician		31	(9.6%)	5	(16.1%)
	veterinary student		74	(22.9%)	3	(4.1%)
	dentist		2	(0.6%)	1	(50.0%)
	medical doctor		19	(5.9%)	2	(10.5%)
	others (non-medical)		159	(49.2%)	26	(16.4%)
owner contact	close		281	(87.0%)	35	(12.5%)
	not close		42	(13.0%)	3	(7.1%)
feeding†	commercial diet		289	(89.5%)	36	(12.5%)
	self-cooked		79	(24.5%)	15	(19.0%)
	raw feeding		45	(13.9%)	4	(8.9%)
Diarrhoea	no		259	(80.2%)	29	(11.2%)
	yes		64	(19.8%)	9	(14.1%)
		acute	34	(10.5%)	6	(17.6%)
		chronic	23	(7.1%)	3	(13.0%)
		acute and chronic	7	(2.2%)	0	(0.0%)
Pre-treatment with antibiotics (previous 4 weeks)	no		269	(83.3%)	24	(8.9%)
	yes		53	(16.4%)	14	(26.4%)
		single	35	(10.8%)	7	(20.0%)
		multiple	13	(4.0%)	5	(38.5%)
		unknown agents	5	(1.5%)	2	(40.0%)
	data not available		1	(0.3%)	0	(0.0%)
Visits to the veterinarian (previous 6 months)	none		103	(31.9%)	8	(7.8%)
	ambulant		193	(59.8%)	24	(12.4%)
	in patient		15	(4.6%)	2	(13.3%)
	ICU		12	(3.7%)	4	(33.3%)

**Immuno-compromised (previous 6 months)**	no		274	(84.5%)	29	(10.6%)
	yes†		46	(14.2%)	8	(17.4%)
		chemotherapy	29	(9.0%)	5	(17.2%)
		illness	26	(8.0%)	5	(19.2%)
		medication	13	(4.0%)	3	(23.1%)
	data not available		3	(0.9%)	1	(33.3%)
**In total**			323		38	(11.8%)

Demographic and clinical data of sampled dogs and numbers of *C. difficile* positive dogs. No statistical significance using $\chi 2$-test could be determined (p-value < 0,05); †groups/subgroups overlapping, only descriptive data analysis
