# Supplementary material for: Dogs as carriers of virulent and resistant genotypes of Clostridioides difficile
Source: Zoonoses Public Health. 2022 May 12;69(6):673–81. doi: 10.1111/zph.12956 (PMC9544694; doi:10.1111/zph.12956)
Supplement: Supplementary file 4 — Table S4 [file ZPH-69-673-s001.pdf]

Table S4 General genomic features of *C. difficile* isolates in this study

ID	bp	contigs	max_c	N50	GC [%]	CDS
35	4136081	487	77319	20234	2878	3667
37	4199581	410	124155	31824	2926	3722
38	4417962	2041	59487	12702	3016	3739
39	4114875	593	73563	16779	2878	3650
47	4238861	455	191182	49464	2904	3755
50	4236857	417	102086	28568	2890	3789
54	4293427	1021	65106	10044	2933	3813
55	4088380	798	65623	11308	2903	3625
62	4150988	910	105200	21353	2904	3592
97	4036865	826	51833	10344	2899	3594
99	4447810	348	186496	60150	2873	4047
108	4633588	3141	60198	15763	3127	3737
112	4168135	248	126886	42752	2852	3800
140	4138666	456	86365	24963	2876	3669
141	4084467	476	66430	18481	2855	3626
142	4437929	313	194058	60231	2876	4051
157	4003864	1071	59321	8625	2911	3458
161	4231811	1535	39442	8187	3017	3645
163	4368965	589	82161	19476	2912	3925
164	4150954	563	113310	21157	2928	3622
169	4361709	664	98631	16762	2930	3951
170	4438860	616	122322	32884	2935	4006
171	4223206	715	99149	22926	2900	3751
173	4090623	310	102778	38568	2858	3638
174	4146322	979	50831	15380	2907	3597
223	4062611	565	109020	41264	2873	3562
225	4068707	228	234606	70934	2856	3559
241	4105486	817	59388	10293	2877	3741
251	4225050	286	167996	54800	2873	3806
272	4238510	679	88674	34898	2898	3742
273	4187387	346	118187	36901	2875	3753
279	4019593	782	57430	10172	2882	3544
308	4265225	1749	44491	9995	3026	3600
316	4302822	296	165988	49893	2891	3838
321	4300349	296	177058	45813	2881	3938
333	4188872	474	76160	23103	2891	3731
337	4203234	266	107961	43385	2857	3853
345	4090548	183	405091	222727	2856	3581
Cd_630	4298133	2	4290252	4290252	2906	3824

ID numbers of isolates, genome size (bp), number of assembled contigs, maximum contig size (max_c), median contig size (N50), GC content and predicted coding sequences (CDS).
